# Root-Knot Nematode (*Meloidogyne incognita*) Control Using a Combination of *Lactiplantibacillus plantarum* WiKim0090 and Copper Sulfate

**DOI:** 10.4014/jmb.2205.05019

**Published:** 2022-07-28

**Authors:** Seulbi Kim, Ho Myeong Kim, Hye Jeong Seo, Jehyeong Yeon, Ae Ran Park, Nan Hee Yu, Seul-Gi Jeong, Ji Yoon Chang, Jin-Cheol Kim, Hae Woong Park

**Affiliations:** 1 Technology Innovation Research Division, World Institute of Kimchi, Gwangju 61755, Republic of Korea; 2 Division of Applied Bioscience and Biotechnology, Chonnam National University, Gwangju 61186, Republic of Korea

**Keywords:** Lactic acid bacteria, root-knot nematode, copper sulfate, nematicidal activity, *Meloidogyne incognita*

## Abstract

Lactic acid bacteria (LAB) exert antagonistic activity against root-knot nematodes, mainly by producing organic acids via carbohydrate fermentation. However, they have not yet been used for root-knot nematode (*Meloidogyne incognita*) control owing to a lack of economic feasibility and effectiveness. In this study, we aimed to isolate organic acid-producing LAB from kimchi (Korean traditional fermented cabbage) and evaluated their nematicidal activity. Among the 234 strains isolated, those showing the highest nematicidal activity were selected and identified as *Lactiplantibacillus plantarum* WiKim0090. Nematicidal activity and egg hatch inhibitory activity of WiKim0090 culture filtrate were dose dependent. Nematode mortality 3 days after treatment with 2.5% of the culture filtrate was 100%, with a 50% lethal concentration of 1.41%. In pot tests, the inhibitory activity of an *L. plantarum* WiKim0090-copper sulfate mixture on gall formation increased. Compared to abamectin application, which is a commercial nematicide, a higher control value was observed using the WiKim0090-copper sulfate mixture, indicating that this combination can be effective in controlling the root-knot nematode.

## Introduction

Root-knot nematodes (*Meloidogyne* spp.) are plant parasites that cause serious economic damage by infecting over 2,000 plant species globally [1]. Second-stage juveniles of *Meloidogyne* spp. invade plant roots and produce galls, thereby disturbing the uptake of nutrients and water by the host plants [2]. The above-ground symptoms of host plants infected by *Meloidogyne* spp. may be similar to those observed in plants with root damage. As specific root-knot symptoms are not apparent above ground, it is difficult to determine the exact moment of outbreak in order to implement crop protection measures. With outdated nematode control methods, host plants could experience heavy root system damage, and this results in economic losses of over $100 billion worldwide [3].

Among more than 100 *Meloidogyne* spp., four major species, namely, *M. incognita*, *M. arenaria*, *M. javanica*, and *M. hapla* are the most common species in agriculture in Korea [4]. These nematodes are problematic owing to their broad host range, high reproductivity, and endoparasitic characteristics [5, 6]. Moreover, continuous cultivation of horticultural crops, such as oriental melon, red pepper, and tomato, makes it difficult to control these nematode species in greenhouses [4, 7, 8].

Chemical nematicides are preferred for nematode control. Although promising as control agents, some have been banned over environmental concerns, residual issues, and toxicity to humans and livestock. Alternative control methods using antagonistic microorganisms such as fungi and bacteria are being investigated [9-11]. As antagonistic microorganisms are not sufficiently efficacious on their own [12], it is necessary to develop a strategy that integrates different control methods to improve the control efficacy.

Lactic acid bacteria (LAB) play a key role in the production of various fermented meat, fish, and dairy products, and kimchi (Korean traditional fermented cabbage) [13]. They are gram-positive bacteria and are divided into two metabolic categories, homofermentative and heterofermentative, based on their metabolism. In general, homofermentative LAB, such as *Lactiplantibacillus* spp., *Lactococcus* spp., and *Pediococcus* spp., are more acid tolerant than heterofermentative LAB, including *Leuconostoc* spp. and *Weissella* spp. The antagonistic activities of homofermentative LAB have been reported against phytopathogenic microorganisms and root-knot nematodes, via the production of organic acids in carbohydrate fermentation [11, 14, 15].

Copper sulfate has been found to be directly toxic to root-knot nematodes in a laboratory test, and it indirectly affects the nematode population under field conditions [16, 17]. When combined with organic acid, copper sulfate shows high synergistic activity against root-knot nematodes [18]. However, only a few studies have been conducted on the nematicidal activity of organic acid-producing LAB combined with copper sulfate.

In the present study, we isolated an organic acid-producing isolate of homofermentative LAB, *Lactiplantibacillus plantarum* WiKim0090, from kimchi. The nematicidal activity of the filtrate of *L. plantarum* WiKim0090 was evaluated against second-stage juveniles (J2) of *M. incognita*. The efficacy of *L. plantarum* WiKim0090 combined with copper sulfate was determined under pot conditions, by investigating gall formation and control value.

## Materials and Methods

### LAB Isolation from Kimchi

LAB were isolated from homemade kimchi obtained from across the Republic of Korea. Five hundred grams of kimchi sample was ground for 2 min using a hand blender. The resulting kimchi juice was filtered through a piece of sterilized cheesecloth, serially diluted with saline solution (0.85%; 3M, USA), and spread on de Man-Rogosa-Sharpe (MRS) agar (Oxoid, England) plates containing CaCO_3_ (2%, w/v). The plates were incubated at 30°C under anaerobic conditions for 2 days, and the tentative LAB strains were selected.

The LAB isolates were cultured anaerobically in MRS broth (Becton, Dickinson and Company, USA) at 30°C for 24 h, transferred to fresh MRS broth (0.1%, v/v), and cultured for another 24 h. Thereafter, the resulting culture was centrifuged at 3,000 *×*g (5810R, fixed angle type; Germany) for 10 min at 4°C. The supernatant was filtered through a 0.20 μm filter, and the filtrate was stored at –20°C for the subsequent experiments.

### Nematode Preparation

An isolate of the root-knot nematode *M. incognita* was obtained from the Korea Research Institute of Chemical Technology, Daejeon, Republic of Korea [11]. Tomato (*Solanum lycopersicum* Mill. ‘Seokwang’) was used as the host plant to maintain *M. incognita*. Tomato root samples infected by the nematode were obtained from a tomato greenhouse at Chonnam National University, and egg masses of *M. incognita* were collected from the infected roots. The obtained egg masses were placed in a solution of 1% sodium hypochlorite, rinsed with distilled water, and used in the experiment [18]. The eggs were incubated for 5 days at 28°C, and freshly hatched J2 juveniles were collected using a Baermann funnel [19].

### In Vitro Nematicidal Activity of LAB Isolates

The mortality of *M. incognita* J2 was determined to evaluate the nematicidal activity of the LAB isolates. Fifty J2 individuals per replicate were placed in 96-well tissue culture plates and treated with the culture filtrate at a concentration of 2.5% [11, 20]. Based on a mortality rate of over 80%, 11 of 234 isolates were selected for further analysis. Four concentrations of the culture filtrate of the LAB isolates showing the highest nematicidal activity (0.31, 0.63, 1.25, and 2.5%) were prepared, and 100 μl of each filtrate was poured into the wells of a 96-well plate. The plates were incubated at 25°C. Mortality was determined after 72 h of treatment, and the motility of J2 individuals was examined. Juveniles that were stiff when touched with a fine needle were considered dead [21]. Sterilized 0.9%saline solution was used as a control. The experiments were repeated three times. Nematicidal activity was calculated as corrected percent mortality [22] and 50% lethal dose (LD50). Mortality was determined as follows:

Mortality (%) = [(*M_a_ − M_b_*) / (100 − *M_b_*] × 100,

where, *M_a_* is the mortality rate in the treatment and Mb is the mortality rate in the control.

To evaluate the egg hatch inhibitory effect of the culture filtrate of WiKim0090, 100 μl of egg solution consisting of 150 *M. incognita* eggs was poured into the wells in a 96-well plate. Five concentrations of the culture filtrate of WiKim0090 (1.25, 2.5, 5.0, 10.0, and 20.0%) were prepared for the treatment. Sterilized 0.9% saline solution was used as a control. The experiments were replicated three times. Egg hatch inhibition was determined 7, 10, and 14 days after treatment, as follows:

Egg hatch inhibition (%) = [(*H_a_ − H_b_*) / (100 − *H_a_*] × 100,

where, *H_a_* is the proportion of J2 individuals in the control, and *H_b_* is the proportion of J2 individuals in the treatment.

### Molecular Identification of WiKim0090

An isolate showing the highest nematicidal activity was identified by 16S rRNA nucleotide sequence analysis. PCR amplification of the 16S rRNA gene was performed using the universal bacterial primer pair 27F (5′-AGAGTTTGATCCTGGCTCAG-3′) and 1492R (5′-GGATACCTTGTTACGACTT-3′). The species was identified by comparing the sequences of related reference strains in the software programs BLASTN and BLASTX of GenBank from the National Center for Biotechnology Information (NCBI).

### Analysis of Organic Acids Produced by WiKim0090

Culture broth of WiKim0090 was centrifuged at 8,000 *×*g (5810R, fixed angle type; Germany), serially diluted, and passed through a 0.45-μm PTFE syringe filter (USA). The amount of organic acids produced was determined by high-performance liquid chromatography (HPLC; Waters Alliance e2695; USA) at 50°C using an Aminex HPX-87H reverse-phase column (300 mm × 7.8 mm, 9 μm particle size; Bio-Rad, USA). Elution was performed isocratically using 5 mmol/L sulfuric acid. The flow rate and detection wavelength were 0.6 ml/min and 210 nm, respectively. Quantitative analysis of the organic acids was performed using standard curves.

### Efficacy of the WiKim0090-Copper Sulfate Mixture on Tomato Plants in Pot Tests

Pots of diameter 9 cm were filled with 100 g of sterile nursery soil–sand mix (1:1, v/v). Four-week-old tomato seedlings (‘Seokwang’) were transplanted into each pot and inoculated with 10 ml of egg solution containing 10,000 eggs. The treatments were formulated as follows: 250- and 500-fold dilutions of 20% copper sulfate pentahydrate (20% CSP) in Tween 20 at 250 ppm (Sigma-Aldrich Co., USA), a 250-fold dilution of WiKim0090 culture broth (WK0090 CB), and 250- and 500-fold dilutions of the mixture of WiKim0090 culture broth and 20%copper sulfate pentahydrate (WK0090 CB + 20% CSP) in Tween 20 at 250 ppm. Tween 20 (250 ppm) was used as a negative control, whereas commercial nematicides, that is, Sunchungtan containing 30% fosthiazate (SL, 4,000-fold dilution; Farm Hannong Co., Korea) and Terra Nova containing 1.68% abamectin (SC, 5,000-fold dilution; Syngenta Korea Co., Korea) were used as positive controls. Twenty milliliters of the treatment solution was poured into each pot. Six weeks after treatment, tomato plants were uprooted, washed to remove the soil, and scored for galls. Galling of the root systems was scored using a 0–5 severity scale [23]. The GI was assigned as: 0 = 1–10%, 1 = 11–20%, 2 = 21–50%, 3 = 51–80%, 4 = 81–90%, and 5 = 91–100% root galls. The control value was determined as follows [24]:

Control value (%) = [(*G_a_−G_t_*) / *G_a_*] × 100,

where, *G_a_* is the galling index for inoculation alone and *G_t_* is the galling index for the treatment.

The experiment was repeated twice with three replicates.

### Statistical Analysis

Analysis of variance and Tukey’s honestly significant difference (HSD) tests were used to determine significant differences among the treatments. Data were analyzed using PASW software (version 17; SPSS Inc., USA).

## Results

### Screening of Nematicidal LAB

**Fig. 1:**
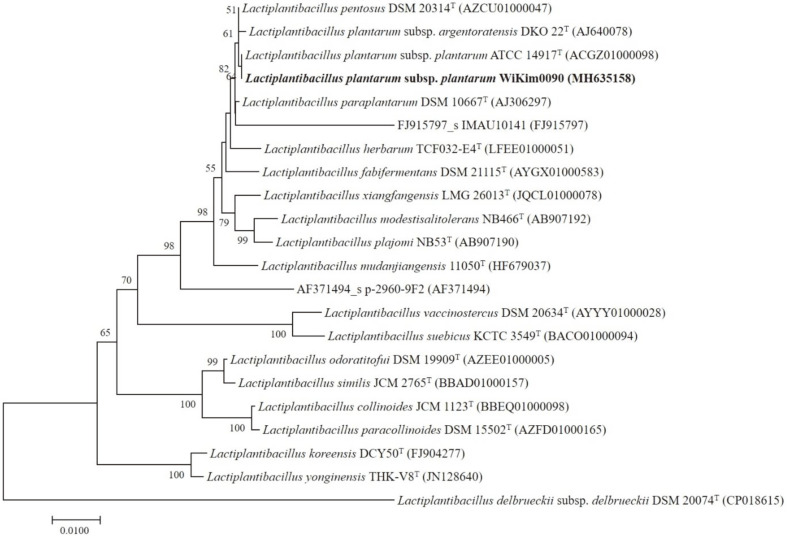
Phylogenetic tree derived from the distance analysis of the 16S rRNA gene sequences of WiKim0090. The sequences were aligned using MEGA 6.0 software. Phylogenetic trees were constructed using the neighbour-joining method with bootstrap analysis (1,000 trials). Bars indicate the percentage of sequence divergence.

Two hundred and thirty-four strains of LAB were isolated from homemade kimchi. Based on the mortality of *M. incognita* J2, WiKim0090, which showed the highest nematicidal activity, was selected and identified as *L. plantarum* (Fig. 1).

### In Vitro Nematicidal Activity of *L. plantarum* WiKim0090

**Fig. 2:**
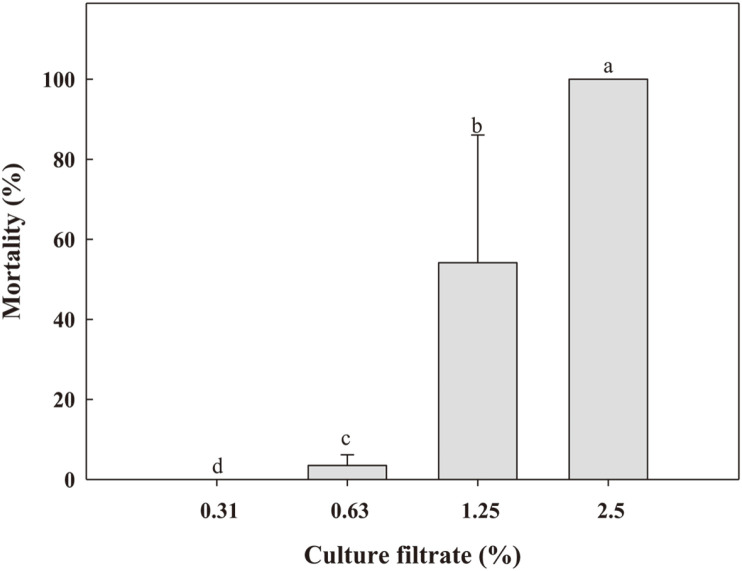
Effect of culture filtrate of *L. plantarum* WiKim0090 on the mortality of second stage juveniles of *M. incognita*. Different letters above the bars indicate that the values are significantly different at *p* < 0.05 (Tukey's honestly significant difference (HSD) test).

**Fig. 3:**
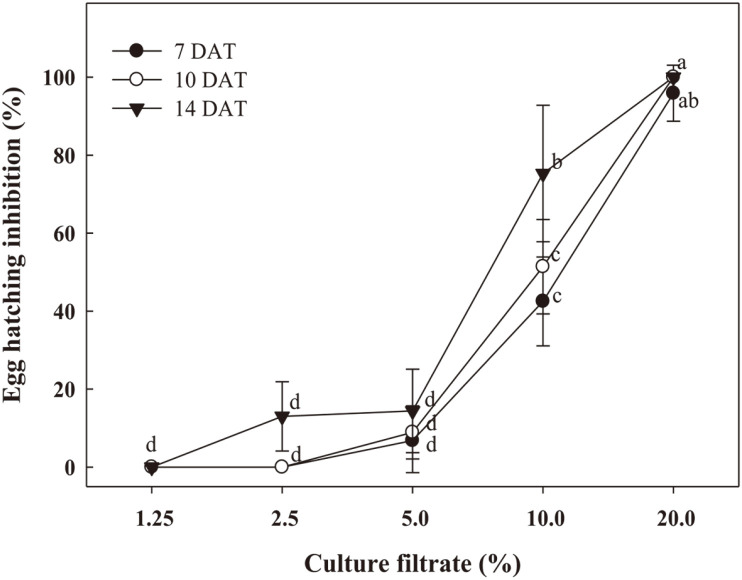
Effect of culture filtrate of *L. plantarum* WiKim0090 on the hatching of eggs of *M. incognita*. Different letters above the symbols indicate values that are significantly different at *p* < 0.05 (Tukey's HSD test).

Nematicidal activity of the culture filtrate of WiKim0090 was dose dependent. The mortality of *M. incognita* J2 increased with the dose from 0.31 to 2.5% (Fig. 2). A mortality rate of 100% was observed 3 days after treatment with 2.5% culture filtrate. The LC_50_ of the Wikim0090 culture filtrate was 1.41%. The egg hatch inhibition rate increased with the culture filtrate concentration (*F* = 266.3; *df* = 4.30; *p* < 0.001); it was also dependent on the exposure time (*F* = 8.8; *df* = 4.30; *P* = 0.001; Table 1). Starting with no inhibition at 1.25%, 42–75% egg hatch inhibition was observed under 10% WiKim0090 culture filtrate treatment (Fig. 3). Compared with the inhibition rate 7 days after treatment (DAT), the rate increased 1.8-fold at 14 DAT. When treated with 20% WiKim0090 culture filtrate, egg hatch inhibition of over 96% was observed at all exposure times.

### Organic Acids Produced by *L. plantarum* WiKim0090

Three kinds of organic acids, namely, acetic acid, citric acid, and lactic acid, were the main metabolites of the WiKim0090 culture filtrate. Acetic acid (4,299 mg/L), citric acid (1,770 mg/L), and lactic acid (17,993 mg/L; Table 2) accounted for 2.4% of acids in the filtrate of WiKim0090.

### Efficacy of the WiKim0090-Copper Sulfate Mixture on Tomato Plants in Pot Tests

**Fig. 4:**
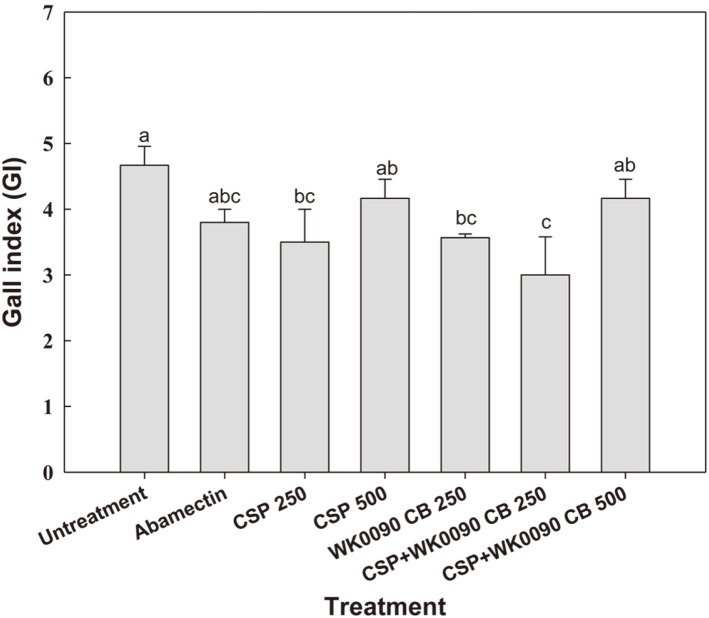
Effects of abamectin, copper sulfate pentahydrate (CSP 250, CSP 500), culture broth of WiKim0090 (WK0090 CB 250), and two combinations of copper sulfate pentahydrate and culture broth (CSP + WK0090 CB 250, CSP + WK0090 CB 500) on root gall formation caused by *M. incognita* in pot tests. Different letters above the bars indicate values are significantly different at *p* < 0.05 (Tukey’s HSD test).

**Fig. 5:**
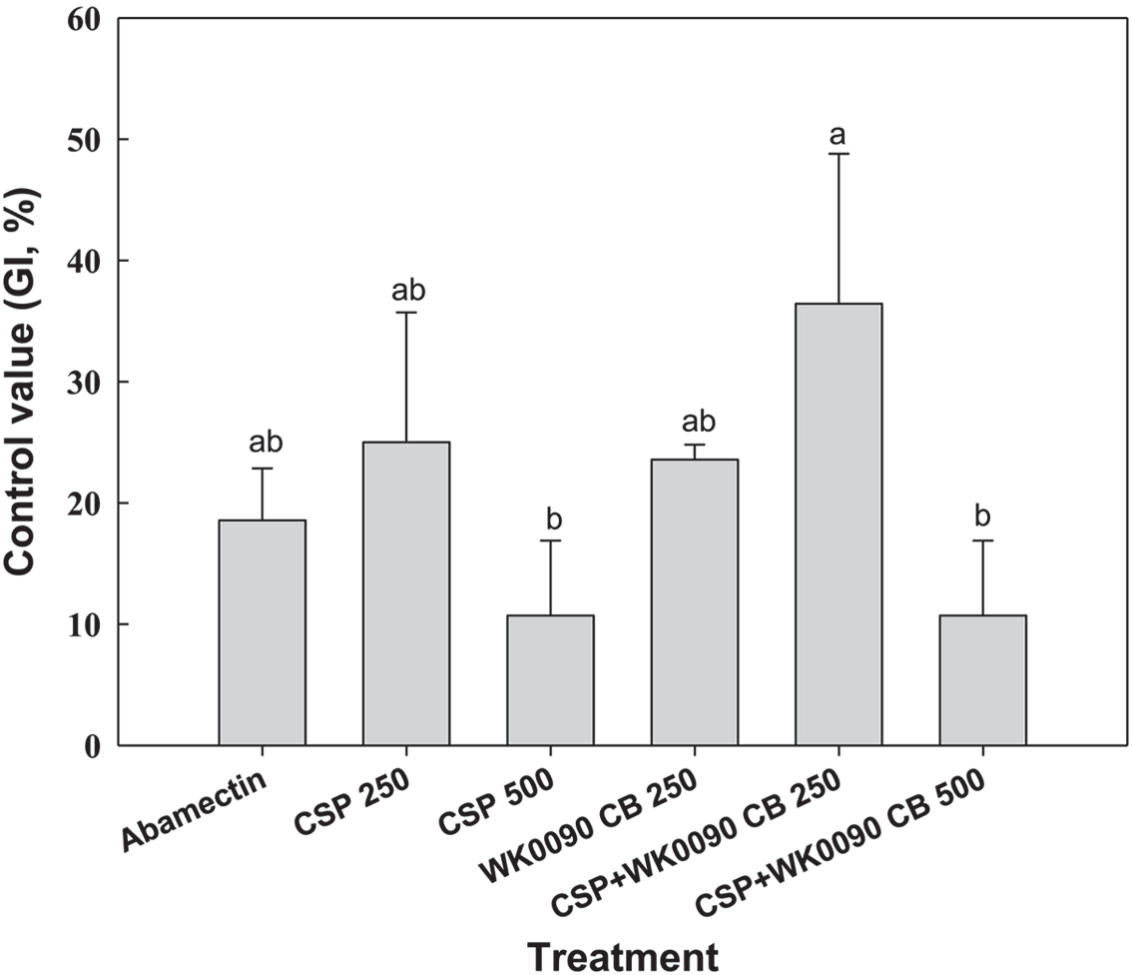
Efficacy of the mixture of WiKim0090 and copper sulfate for biological control of *M. incognita* in pot experiments. Different letters above the bars indicate values that are significantly different at *p* < 0.05 (Tukey’s HSD test). CSP, copper sulfate pentahydrate; WK0090 CB, culture broth of WiKim0090; CSP + WK0090 CB, combination of copper sulfate pentahydrate and culture broth.

A significant difference in gall index was observed between treated and non-treated groups (*F* = 39.2; *df* = 7,16; *p* < 0.001). The efficacy of 250-fold dilution of copper sulfate was comparable with that of the 250-fold dilution of WK0090 CB and the 5,000-fold dilution of abamectin, with galling index (GI) of 3.5, 3.6, and 3.8, respectively (Fig. 4). When WK0090 CB was mixed with CSP, the inhibitory activity of the 250-fold dilution mixture on gall formation further increased with a GI of 3.2, whereas the untreated control showed the highest GI of 4.7. The highest control value of 36.4% was observed after treatment with a 250-fold dilution of WK0090 CB + 20% CSP, followed by a 250-fold dilution of 20% CSP and a culture broth of WiKim0090, with control values of 25.0 and 23.1%, respectively (Fig. 5).

## Discussion

The development of bionematicides using antagonistic microorganisms has been of interest for sustainable pest management in greenhouse cultivation [10, 25-27]. Among the antagonistic microorganisms, LAB have been extensively studied to protect plants against not only phytopathogenic microorganisms, such as *Colletotrichum capsici*, *Erwinia carotovora*, and *Xanthomonas campestris*, but also root-knot nematodes [15, 28]. LAB from vegetable products produce various organic acids from carbohydrates, which contribute to the inhibition of nematode viability and egg hatching [11].

*Lactiplantibacillus plantarum* Wikim0090 produced lactic acid as the main metabolite in a submerged culture, followed by acetic acid, and citric acid. Exposure to these organic acids results in the destruction of tissues and cells in the outer surfaces of nematodes and their eggs [29]. Both nematode mortality and egg hatch inhibition caused by *L. plantarum* WiKim0090 were lower than those caused by *L. brevis* WiKim0069 as determined in our previous study [11]. The total organic acid concentration produced by both strains was similar, but the acid profiles were different. Acetic acid was the main organic acid in WiKim0069, whereas lactic acid was the main acid in WiKim0090. These results indicate that the profile and concentrations of these acids may have an effect on nematicidal activity. Acetic acid and lactic acid showed strong nematicidal activity when used separately at a concentration of 0.2% [24]. A mixture of acetic acid and lactic acid presented increased nematicidal activity at a lower concentration of organic acids, as the activity was largely attributable to acetic acid.

The biocontrol efficacy of the antagonistic microorganisms *Bacillus firmus*, *Myrothecium roridum*, and *Verticillium lecanii* showed a positive correlation with the dilution of microbial culture broth [10, 30, 31]. Excessive dilution of microbial culture broth substantially lowers biocontrol efficacy, causing difficulties in application under field conditions. These results indicate that sufficient concentrations of active ingredients in the microbial culture broth are needed to ensure biocontrol effectiveness. This was the case in our study—a substantial amount of organic acids and microbial primary metabolites were produced and contained in WiKim0090 culture broth. A total concentration of organic acids of 2.4% may be efficacious in pot tests. A 250-fold dilution of WiKim0090 culture broth effectively suppressed gall formation in the pot test, showing biocontrol efficacy comparable with that of the commercial nematicidal agent, abamectin. The egg hatch rate of *M. incognita* showed a negative correlation with the concentration of lactic acid from 5 to 25 μg/ml [32]. This might have accounted for the biocontrol effect in the pot tests, considering that the lactic acid concentration was approximately 100 μg/ml in the 250-fold dilution of WiKim0090 culture broth. The high dilution rate of microbial culture broth may be cost effective, and thereby accelerate the commercial production of bionematicides using LAB.

Copper sulfate was used as an effective control agent against a gastrointestinal nematode species (*Haemonchus contortus*) found in sheep in the early 1900s [33]. It reduces gall formation by *M. incognita* in the roots of garden beets and tomatoes and shows synergistic nematicidal activity when applied together with maleic acid [18, 34]. The weight of tomato roots and shoots increased with the application of copper sulfate. However, excessive use of copper sulfate is toxic to living creatures and causes metal contamination in soil, although copper is necessary for plant growth [35]. In our study, the control value of gall formation increased with the increase in copper sulfate concentration in the pot test. When treated with WiKim0090 culture broth, the control value of the mixture further increased in pot tests, probably because of the continuous production of organic acids by WiKim0090. As low-molecular-weight organic acids, including acetic acid and lactic acid, are known to remain in the soil for only a short period, the biocontrol efficacy attributable to these organic acids may not be long-lasting [18, 36, 37]. Therefore, organic acids alone are not actively used for root-knot nematode control. Organic acid-producing WiKim0090 was used to solve this problem because WiKim0090 may continue to produce and supply organic acids to the soil. Moreover, we found that the combination of organic acid-producing WiKim0090 and copper sulfate resulted in a decrease in the amount of copper sulfate used.

*Lactiplantibacillus plantarum* WiKim0090, on its own, is not considered a biological control agent despite its nematicidal activity and inhibitory effect on egg hatching. However, the mixture of copper sulfate pentahydrate and WiKim0090 culture broth clearly showed biological control potential with respect to suppression of gall formation and efficacy in the pot. In addition, the practical dilution rate of the mixture could accelerate its commercial production. Further processes, such as a formulation strategy for biocontrol efficacy and long-term storage, elucidation of the mechanism of the product, and investigation of potential harmful effects on the hosts and soil environment, are required prior to the commercialization of the mixture of LAB and copper sulfate pentahydrate as a suitable nematicide.

## Figures and Tables

**Table 1:** Analysis of variance results for the effect of culture filtrate on the hatching of eggs of *Meloidogyne incognita*.

	df	SS	MS	*F* value	*P* value
Exposure time	2	1,087.9	544.0	8.8	0.001
Concentration	4	65,682.9	1,6420.7	266.3	0.000
Time × Concentration	8	1,123.1	140.4	2.3	0.49
Residuals		1,849.8	61.7		

df: degree of freedom, SS: sample size, MS: mean square

**Table 2:** Organic acids produced by *L. plantarum* WiKim0090 in Lactobacilli de Man-Rogosa-Sharpe broth.

Organic acid	Yield (μg/ml)
Acetic acid	4,299
Citric acid	1,770
Fumaric acid	ND^a^
Lactic acid	17,993
Malic acid	ND^a^
Malonic acid	ND^a^
Oxalic acid	ND^a^
Succinic acid	ND^a^
Tartaric acid	ND^a^

^a^ND, not detected
